# Synthesis of a Highly Luminescent Three-Dimensional Pyrene Dye Based on the Spirobifluorene Skeleton

**DOI:** 10.3390/molecules15117582

**Published:** 2010-10-28

**Authors:** Kentaro Sumi, Gen-ichi Konishi

**Affiliations:** Department of Organic and Polymeric Materials, Graduate School of Science and Engineering, Tokyo Institute of Technology, Japan

**Keywords:** Spirobifluorene, pyrene, fluorescence, π-conjugation, dye

## Abstract

We have synthesized a highly luminescent (log ε > 5.0, Φ > 0.9) pyrene dye based on a spirobifluorene skeleton [2,2′,7,7′-tetrakis(7-*tert*-butyl-1-pyrenyl)-9,9′-spirobi[9H-fluorene; **4-PySBF**]. The use of spirobifluorene prevents fluorescence quenching by intramolecular energy transfer and/or electron transfer among the chromophores in the excited state. The emission spectra of **4-PySBF** exhibited a red shift of 20 nm in comparison to a model compound [9,9′-dioctyl-2,7-bis(7-*tert*-butyl-1-pyrenyl)-9H-fluorene; **2-PyF**], but its UV-Vis spectrum remained unchanged.

## Introduction

Recently, blue emitting dyes with high efficiency (Φ ≈ 1.0, log ε > 4.5) [1] have been attracting considerable attention because of their applicability to molecular electronic materials such as organic field effect transistors (OFETs) and organic light-emitting diodes (OLEDs). Many studies have been conducted on strategies for synthesizing a highly efficient dye. For example, rod- [1,2] and star-shaped [3,4,5,6] hydrocarbons that expand π-conjugation to two dimensions can be used to control the emission color of a dye with high efficiency.

Spirobifluorene derivatives are considered the most promising candidates for organic optoelectronics [7,8,9,10,11]. The rigidity of spiro-compounds affords them high thermal stability. In addition, their solubility is higher than that of corresponding compounds without a spiro moiety, because their perpendicular conformations that are based on the spiro-linkage efficiently suppress intermolecular interactions between π-systems. Recently, several studies were conducted on introducing dyes into a molecule for π-conjugation in three dimensions. Examples of such studies include those on the introduction of dyes into the spirobifluorene skeleton [12,13,14,15,16,17,18,19]. Because of the high thermal stability and unique characteristics of the spirobifluorene skeleton, it has been adopted in various materials [20,21,22]. 

In this paper, we report the synthesis and characterization of a new pyrene dye that is based on the spirobifluorene skeleton. Pyrene derivatives are highly absorptive [23,24,25,26]; further, the introduction of pyrene substituents into spirobifluorene derivatives is expected to improve the fluorescence quantum yield and thermal stability of the derivatives because the substituents are highly emissive, bulky, and rigid. 

## Results and Discussion

**Scheme 1:**
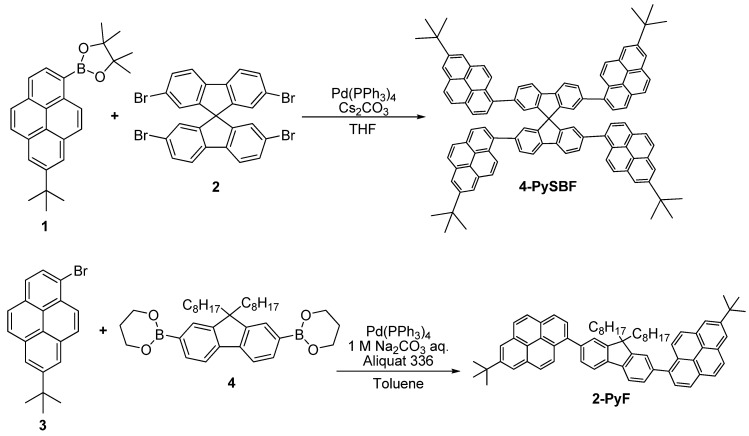
Synthesis of spirobifluorene dye and model compound.

A spirobifluorene dye, 2,2′,7,7′-tetrakis(7-*tert*-butyl-1-pyrenyl)-9,9′-spirobi [9H-fluorene] (**4-PySBF**), and a model compound, 9,9′-dioctyl-2,7-bis(7-*tert*-butyl-1-pyrenyl)-9H-fluorene (**2-PyF**), were synthesized in 37% and 24% yields, respectively, by palladium-catalyzed Suzuki-Miyaura coupling reactions (Scheme 1). The precursors, 7-*tert*-butylpyrene-1-boronic acid pinacol ester (**1**) and 1-bromo-7-*tert*-butylpyrene (**3**), were prepared from pyrene according to a previously reported method [27]. The chemical structures of **4-PySBF** and **2-PyF** were confirmed by ^1^H-NMR, ^13^C-NMR, and FT-IR spectroscopy. Each compound was found to be soluble in various organic solvents such as chloroform, tetrahydrofuran (THF), dichloromethane, and toluene. It should be noted that the *tert*-butyl group is necessary for the successful synthesis of **4-PySBF**; we found that without *tert*-butyl groups, the solubility is quite poor, making purification and structural analysis by techniques such as ^1^H-NMR and ^13^C-NMR rather difficult.

We measured the UV-Vis spectra, fluorescence spectra, fluorescence quantum yields, and fluorescence lifetimes, and lifetimes of **4-PySBF** and **2-PyF** in CH_2_Cl_2_ solution. The UV-Vis spectra (Figure 2, left side) show that the absorption maxima (λ_max_) of **4-PySBF** and **2-PyF** are at 360 nm and 359 nm, with molar absorption (λ_max_) coefficients ε of 131,000 and 71,000 M^−1^ cm^−1^, respectively. The finding that the ε value of **4-PySBF** is about twice that of **2-PyF** is in accordance with the fact that **4-PySBF** has two chromophores per molecule whereas **2-PyF** has but one.

The observed fluorescence spectra (Figure 2, right side) show that **4-PySBF** and **2-PyF** exhibit intense blue emissions (λ_em_) at 441 nm (Φ = 0.92) and 421 nm (Φ = 0.90), respectively, in CH_2_Cl_2_ solution (λ_ex_ = 360 nm; *c* = 1.0 × 10^−6^ M). The fluorescence spectra also reveal that the fluorescence intensity of **4-PySBF** is about twice that of **2-PyF**, again because **4-PySBF** has two chromophores per molecule. A 20-nm red-shift between **4-PySBF** and **2-PyF** was observed. It is noted that we observed a spiroconjugation (a large red shift in the fluorescence spectrum and no red shift in the absorption spectrum in this **4-PySBF** and **2-PyF** pair). We hypothesize that this observed red-shift could be due to conjugation of **4-PySBF** *via* the spiro carbon [28,29,30,31,32] in the excited state [33]. Another possibility is a through-space interaction between upper and lower pyrene chromophores.

**Figure 1:**
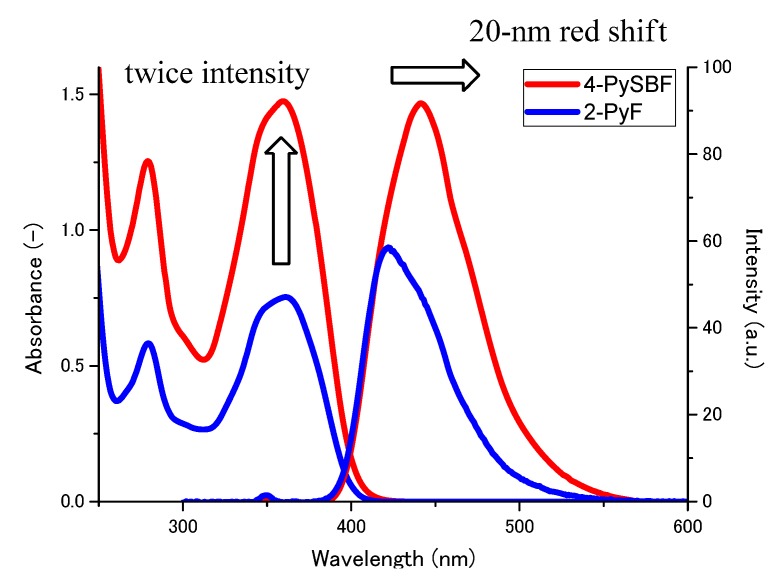
Absorption and emission spectra of **2-PyF** and **4-PySBF** in CH_2_Cl_2_; *c* = 10 μM, λ_ex_ = 360 nm.

**Table 1:** Photochemical data for compounds **4-PySBF** and **6**.

**dye**	λ_ab_[nm]	ε (×10^5^) [l/mol･cm]	log ε	λ_em_[nm]	Φ	τ [ns]	*k*_f_ (×10^8^) [S^-1^]	*k*_isc_ + *k*_nr_ (×10^7^) [S^-1^]
**4-PySBF**	360	1.31	5.12	441	0.92	1.52	6.1	5.3
**2-PyF**	359	0.71	4.85	421	0.90	1.88	4.8	5.3

Measurement of the photophysical properties of both compounds at the same concentration confirm that **4-PySBF** had twice the number of chromophores in solution as compared to **2-PyF**. Next, we measured the fluorescence spectra of both compounds under the same absorption conditions. We then found that the solutions of **4-PySBF** and **2-PyF** had the same number of chromophores. The fluorescence spectra (Figure 3) revealed that both **4-PySBF** and **2-PyF** had approximately the same fluorescence intensity. This result shows that the spirobifluorene derivatization doubled the performance of the chromophores by preventing intramolecular energy transfer and/or electron transfer, although the pyrene chromophores are placed close together. The compound **4-PySBF** was found to have ideal luminescence properties (log ε > 5.0, Φ > 0.9).

**Figure 2:**
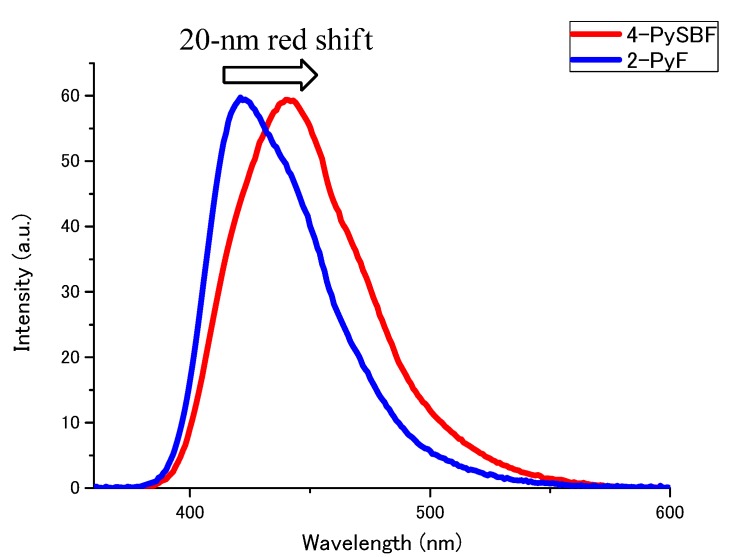
Emission spectra of **2-PyF** and **4-PySBF** in CH_2_Cl_2_; Abs = 0.1, λ_ex_ = 360 nm.

Fluorescence quantum yield can be expressed as Φ_f_ = *k*_f_τ_s_, or Φ_f_ = *k*_f_/(*k*_f_ + *k*_isc_ + *k*_nr_ + *k*_q_[O_2_]), where *k*_f_, *k*_isc_, *k*_nr_, and *k*_q_[O_2_] denote the rate constants for fluorescence radiation, intersystem crossing, nonradiative decay, and fluorescence quenching by oxygen, respectively, and τ_s_ denotes the lifetime (s = singlet) [14]. Table 1 summarizes the values of *k*_f_ and *k*_isc_ + *k*_nr_ for **4-PySBF** and **2-PyF**, calculated from the experimental values of Φ_f_ and τ_s_ under degassed conditions, that is, under the assumption that *k*_q_[O_2_] = 0. As *k*_nr_ for aromatic hydrocarbons is known to be negligible [34], *k*_isc_ should be the dominant component in *k*_isc_+*k*_nr_ [35,36,37,38]. Under aerated conditions, *k*_q_[O_2_] is estimated to be nearly diffusion controlled (up to 3.89 × 10^10^ m^−1^ s^−1^ in hexane) [34,39], and [O_2_] is less than 2.0 × 10^−3^ M in usual solvents. These data imply that the maximum possible value of *k*_q_[O_2_] is 8.0 × 10^7^ s^−1^. As can be seen from Table 1, the *k*_f_ values for **4-PySBF** and **2-PyF** are much larger than the *k*_q_[O_2_] values. This situation is most probably responsible for the observed large values of Φ_f_ for both compounds, even in the presence of oxygen.

## Conclusions

In summary, we have synthesized a highly luminescent spirobifluorene dye (log ε > 5.0, Φ > 0.9) with tetrapyrene chromophores (**4-PySBF**) and measured its photophysical properties. In comparison to a model compound, **2-PyF**, the emission spectrum of **4-PySBF** exhibited a red shift of 20 nm, but its UV-Vis spectrum remained unchanged. It is not easy to find a satisfactory explanation for this observed red-shift at the present stage. However, in our next study, we intend to calculate the electron states of these compounds in the ground and excited states by the molecular orbital (MO) method and examine this conjugation system in greater detail. Further, we intend to apply **4-PySBF** in a previously developed cholesteric liquid crystal laser (distributed feedback laser) as the laser dye in the future.

## Experimental

### Instruments

All the ^1^H- and ^13^C-NMR spectra were recorded on a 400 MHz JEOL LMN-EX400 instrument with tetramethylsilane (TMS) as the internal standard. FT-IR spectra were recorded on a JASCO FT-IR 469 plus spectrometer. Melting points were obtained by a Stuart Scientific Melting Point Apparatus SMP3. MS spectra (FAB) were obtained by JEOL JMS700 mass spectrometer. UV-Vis spectra were recorded with a Beckman Coulter DU800 UV-Vis Spectrophotometer. Fluorescence spectra were recorded on a JASCO FP-6500 Spectrofluorometer. Quantum Yields were measured by a Hamamatsu Photonics C9920-02 Absolute PL Quantum Yield Measurement system. Fluorescence lifetimes were measured using a Hamamatsu Photonics OB 920 Fluorescence Lifetime Spectrometer. 

### Materials

Unless otherwise noted, all reagents, chemicals and solvents were obtained from commercial sources and used without further purification. Pyrene, 2-chloro-2-methylpropane, Pd(PPh_3_)_4_, 4,4,5,5-tetramethyl-1,3,2-dioxaborolane were obtained from TCI. 9,9-Dioctyl-fluorene-2,7-bis(trimethylene-borate) (**4**) and PdCl_2_(PPh_3_)_2_, were obtained from Aldrich. 2,2′,7,7′-Tetrabromospirobifluorene (**2**) was a gift of JFE Chemical Coporation (Japan).

*2,2′,7,7′-tetrakis(7-tert-Butyl-1-pyrenyl)-9,9′-spirobi [9H-fluorene]* (**3**, **4-PySBF**)

Under an argon atomosphere, 2,2′,7,7′-tetrabromospirobifluorene (0.25 g, 0.4 mmol), 7-*tert*-butylpyrene-1-boronic acid pinacol ester (0.77 g, 2.0 mmol) and cesium carbonate (5.21 g, 16 mmol) were mixed together with Pd(PPh_3_)_4_ (100 mg, 0.1 mmol) and degassed THF (30 mL). The mixture was refluxed for 24 h. After cooling to room temperature, the resulting mixture was extracted with chloroform. The organic extract was washed sequentially with water and brine and then dried over MgSO_4_. After removal of the solvent, the residue was purified by column chromatography (chloroform:hexane = 1:5) and preparative HPLC (CHCl_3_). After removal of the solvent, the product was recrystallized from cyclohexane to afford **4-PySBF** as a yellow powder in 37% yield. ^1^H-NMR (400 MHz, THF-d_8_): δ 8.23–7.36 (m, Ar-H, 44H), 1.53 (s, *tert*-butyl-H, 36H) ppm.; ^13^C-NMR (100 MHz, CDCl_3_): δ 149.3, 149.1, 140.9, 140.7, 137.4, 131.3, 130.7, 130.5, 130.3, 128.4, 128.2, 128.1, 127.7, 127.5, 127.3, 127.2, 126.5, 125.0, 124.9, 124.4, 123.1, 122.4, 122.0, 120.2 (aromatic C), 66.3 (spiro-C), 35.2 (Ar-C (CH_3_)_3_), 31.9 (Ar-C (CH_3_)_3_) ppm; FT-IR (KBr): 3042, 2961, 2901, 2867, 1594, 1457, 1227 cm^−1^; mp 286–288 °C; HR-MS (FAB^+^) Calcd. for C_105_H_80_ [M] 1341.6294, found [M^+^] 1341.6290. 

*9,9′-Dioctyl-2,7-bis (7-tert-butyl-1-pyrenyl)-9H-fluorene* (**6**, **2-PyF**)

Under an argon atomosphere, 2-bromo-7-*tert*-butylpyrene (0.8 g, 2.4 mmol) and 9,9-dioctyl-fluorene-2,7-bis(trimethyleneborate) (0.28 g 0.5 mmol) were mixed together with Pd(PPh_3_)_4 _(60 mg, 0.05 mmol), degassed toluene (20 mL), one drop of Aliquat 336, and 1M aqueous sodium carbonate solution (2 mL). The resulting mixture was refluxed for 24 h. After cooling to room temperature, the mixture was extracted with chloroform. The organic extract was washed sequentially with water and brine and then dried over MgSO_4_. After removal of the solvent, the residue was purified by column chromatography using chloroform/hexane (5:1, v/v) following HPLC (CHCl_3_). After subsequent recrystallization in ethanol, **2-PyF** was obtained as a yellow powder in 24% yield. ^1^H-NMR (400 MHz, CDCl_3_): δ 8.27–7.97 (m, Ar-H, 18H), 7.69–7.67 (m, Ar-H, 4H), 2.12–2.08 (s, Ar-CH-Ar, 2H), 1.60 (s, *tert*-butyl-H, 18H), 1.25–1.19 (m, alkyl-H, 20H), 0.96 (m, alkyl-H, 4H), 0.82 (t, *J* = 6.84, alkyl-H, 6H) ppm; ^13^C-NMR (100 MHz, CDCl_3_): δ 151.2, 149.1, 140.1, 140.0, 138.1, 131.4, 130.9, 130.4, 129.4, 128.4, 127.6, 127.5, 127.3, 125.4, 125.3, 125.0, 124.5, 123.2, 122.4, 122.0, 119.8 (aromatic C), 55.3 (spiro-C), 40.4 (alkyl-C), 35.2 (Ar-C (CH_3_)_3_), 31.9 (Ar-C (CH_3_)_3_), 30.1, 29.7, 29.4, 29.3, 24.2, 22.7, 14.2 (alkyl-C) ppm; FT-IR (KBr): 3050, 2953, 2925, 2853, 1608, 1481, 1457, 1310 cm^−1^; mp 206–208 °C; HR-MS (FAB^+^) calcd. for C_69_H_74_ [M] 902.5791, found [M^+^] 902.5786. 

## Figures and Tables

**Figure S1:**
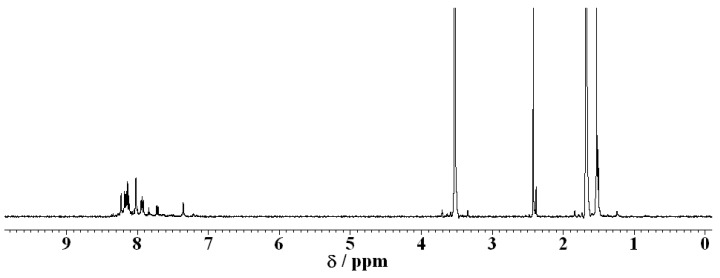
^1^H-NMR spectrum of 2,2′,7,7′-tetrakis(7-*tert*-butyl-1-pyrenyl)-9,9′-spirobi [9H-fluorene] (**4-PySBF**, 400 MHz, THF-d_8_).

**Figure S2:**
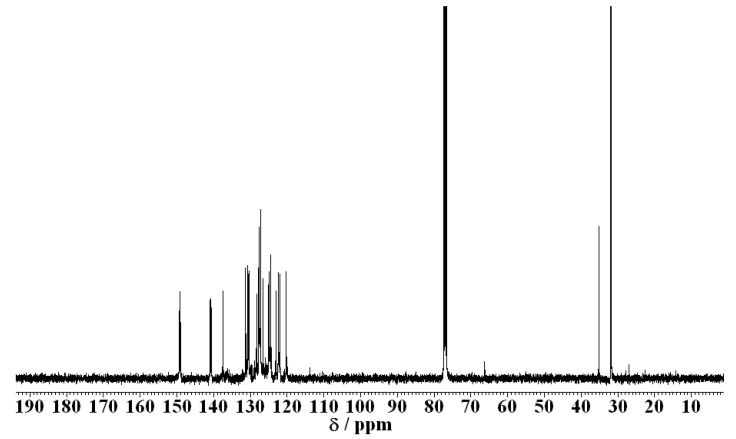
^13^C-NMR spectrum of 2,2′,7,7′-tetrakis(7-*tert*-butyl-1-pyrenyl)-9,9′-spirobi [9H-fluorene] (**4-PySBF**, 100 MHz, CDCl_3_).

**Figure S3:**
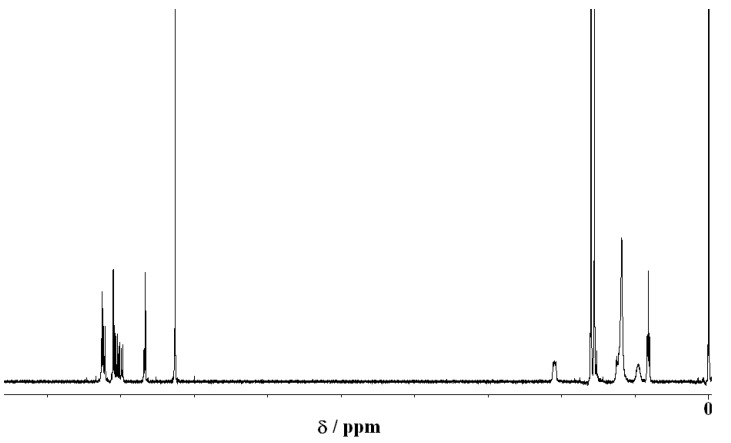
^1^H-NMR spectrum of 9,9′-dioctyl-2,7-bis (7-*tert*-butyl-1-pyrenyl)-9H-fluorene (**2-PyF**, 400 MHz, CDCl_3_).

**Figure S4:**
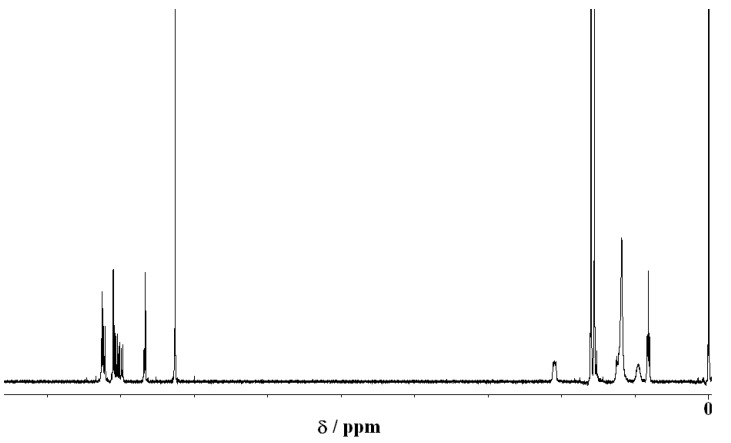
^13^C-NMR spectrum of 9,9′-dioctyl-2,7-bis (7-*tert*-butyl-1-pyrenyl)-9H-fluorene (**2-PyF**, 100 MHz, CDCl_3_).

## Appendix

^1^H-NMR and ^13^C NMR spectra of **3** and **6**
